# Integrated Multidisciplinary Treatment for Pediatric Inflammatory Bowel Disease

**DOI:** 10.3390/children8020169

**Published:** 2021-02-23

**Authors:** Anava A. Wren, Michele H. Maddux

**Affiliations:** 1 Department of Pediatrics, Division of Gastroenterology, Hepatology, and Nutrition, Stanford Children’s Inflammatory Bowel Disease Center, Stanford University School of Medicine, Stanford, CA 94304, USA; 2 Division of Developmental and Behavioral Health, Children’s Mercy Kansas City, Kansas City, MO 64108, USA; mhmaddux@cmh.edu; 3 Division of Gastroenterology, Children’s Mercy Kansas City, Kansas City, MO 64108, USA; 4 Department of Pediatrics, University of Missouri Kansas City School of Medicine, Kansas City, MO 64110, USA

Inflammatory Bowel Disease (IBD) is a chronic and relapsing inflammatory disorder of the gastrointestinal tract. Crohn’s Disease (CD) and Ulcerative Colitis (UC) are the two most common forms of IBD, marked by periods of disease flare and remission. IBD is affecting a growing number of children, with peak onset occurring during adolescence. In the United States, the incidence of pediatric IBD is approximately 10 out of 100,000 children [1]. As the prevalence of pediatric IBD rises, so does the importance and urgency of better understanding and holistically treating IBD.

The pathophysiology of IBD is thought to be multifactorial and involve a complex interaction between genetic factors, the immune system, gut microbiota, nutrition, and psychosocial factors. A deeper understanding of the connection between the brain and the gut has also shed light on how the brain–gut axis may impact IBD. Research has demonstrated that psychological factors can influence the gut through the central nervous system, and modulation of gut stimuli and hypothalamic–pituitary–adrenal axis, and the gut is thought to impact the brain primarily via the gut microbiota-inflammatory pathway [2,3]. Of note, studies have demonstrated that psychosocial factors (e.g., depression) have been shown to be strong predictors of negative health outcomes in pediatric IBD, such as disease activity and risk of relapse, as well as nonadherence, healthcare utilization, and higher health care costs [4,5]. There is also preliminary evidence that inflammatory activity is associated with the development of psychiatric disorders [6,7]. Additionally, children with IBD are at increased risk for emotional/behavioral challenges and poorer quality of life compared to healthy children and youth with other chronic health conditions, and these challenges have shown strong links to poor health outcomes [8,9,10,11]. While additional research is needed to better understand the complex bidirectional relationship between inflammatory activity and psychological functioning in IBD, studies to date have highlighted that children with IBD are a high-risk population that could benefit from integrated care addressing intersecting biological, psychological, and social needs.

An integrated multidisciplinary care model for the treatment of pediatric IBD joins gastroenterologists with mental health providers and other subspecialties to provide comprehensive patient care from a biopsychosocial perspective. Each provider is recognized as an expert and collaborator who is vital in providing patient treatment. The goal and benefit of such holistic treatment plans is that providers work jointly, and in parallel, with each other and families to prioritize disease management, functioning across domains, and the wellbeing of children with IBD at all stages of disease. This care model is increasingly common and deemed essential for optimal care. Multidisciplinary treatment teams that combine the expertise of gastroenterologists, surgeons, nurses, dieticians, psychologists, and social workers collaboratively develop individualized and comprehensive treatment plans that support the whole child and family.

One size does not fit all in integrated multidisciplinary care models. What works for one pediatric practice may not be successful for another pediatric practice. Clinicians are encouraged to individualize integrated care models to the specific needs of patients, with consideration given to the specific setting, patient population, and resources of the practice. One example of an integrated health care model is the IBD Clinic at Children’s Mercy-Kansas City, established in 2011. This clinic uses a multidisciplinary team approach in the assessment and treatment of children and adolescents with IBD, and is staffed by medical, psychology, and affiliated healthcare professionals (e.g., dietician, social worker, pharmacist) most of whom are dedicated entirely to this patient population. Patients are seen by the interdisciplinary team within one to four weeks of their initial IBD diagnosis with once yearly follow-up thereafter with the team for continuity of care. Routine medical care between these yearly visits to the IBD Clinic is provided by each patient’s primary gastroenterologist. As part of the standard clinical care during these visits, patients and caregivers complete psychosocial questionnaires on the patient’s emotional/behavioral functioning, quality of life, and adherence. This routine screening allows the team to monitor a patient’s psychosocial functioning over time and proactively target any functional changes before they develop into clinically significant psychosocial difficulties. Another integrated IBD clinic at Children’s Mercy-Kansas City, which began in 2017, is a Young Adult IBD Clinic. It is staffed by a multidisciplinary team comprised of an APRN, pharmacist, psychologist and social worker, and is focused on transition planning, self-management and self-advocacy among young adult patients. As part of standard practice in this clinic, patients and caregivers complete questionnaires on self-efficacy, transition readiness, perceived transition barriers, and allocation of treatment responsibility. This information is reviewed by the team collaboratively and used to guide the content of the visit. Patients are seen in the Young Adult IBD Clinic one to four times per year, depending on the patient’s age and transition preparedness.

Stanford Children’s IBD Center is another program that employs an integrated team approach in the evaluation and treatment of children with IBD. The primary team is comprised of IBD gastroenterologists, nurses/nurse practitioners, and a psychologist, social worker, dietician, and research and clinic coordinator. Since 2017, newly diagnosed patients have been seen by the IBD team approximately one to three months after diagnosis to provide a comprehensive evaluation and determine the various services that children and families may need, from medical to psychosocial and nutritional support. Patients and caregivers also complete medical, psychosocial, and dietary questionnaires at this visit to assess important IBD-related outcomes and highlight any clinical or functional challenges that should be a target of the visit, as well as patients’ strengths and wellbeing. While patients are primarily followed by an IBD gastroenterologist or nurse practitioner, many patients and families continue to receive integrated IBD treatment and support from a dietician, social worker, and/or psychologist. Common examples of this integrated care include supporting a child who is struggling with mood, anxiety, or adjustment challenges related to IBD, nonadherence to medical and/or dietary therapies, or persistent pain. Additionally, Stanford Children’s IBD Center has specialized interdisciplinary clinics focused on IBD integrative medicine, IBD nutrition, and IBD transition (from pediatric to adult care) staffed by medical providers and affiliated healthcare providers.

Our colleagues, who have similarly developed notable integrated IBD programs at their respective institutions, are highlighted in this Special Issue.

Building an integrated pediatric IBD program is an iterative and thoughtfully constructed process. Our collaborative endeavors in pediatric IBD care have highlighted that one of the most frequently reported challenges when building an integrated IBD program is the absence of multidisciplinary care providers, most notably psychologists and social workers. In fact, the question, “How do we secure psychosocial providers on our team?” is a common and important one. Addressing this issue often necessitates a process of demonstrating the value added by integrating psychosocial providers in the routine care of young patients with IBD. This might involve a period of data collection to show the unmet needs of patients in the absence of a psychologist and social worker; for example, the proportion of patients with psychosocial needs who cannot easily access necessary resources in the community, the impact of such unmet psychosocial needs on IBD-related health outcomes, and the amount of time providers spend addressing psychosocial needs during routine visits. Of equal importance is the process of demonstrating the positive impact of integrated psychosocial care on health outcomes and drawing on the research base to strengthen this argument. Such data can be invaluable when leveraging institutional support to secure psychologists and social work providers in the care of IBD patients.

Training the next generation of pediatric gastroenterologists to practice within a biopsychosocial framework is an approach that warrants strong consideration and has relevance to practice across the developmental lifespan of a gastroenterology professional. Curricula for education and training should be developed to incorporate behavioral medicine into GI fellowship training (i.e., integration of behavioral, psychosocial, and biomedical science knowledge in the prevention, diagnosis, and treatment of illness), meet key competencies, and address behavioral-medicine-relevant information in the required American Board of Pediatrics board exams. It is widely recognized that integrated care can improve patients’ quality of life and health outcomes, as well as reduce overall health care costs and utilization. The North American Society for Pediatric Gastroenterology, Hepatology, and Nutrition (NASPGHAN) has published training guidelines that emphasize the importance of a biopsychosocial paradigm and integrated care [12], but such models have not yet emerged as the practice standard. As such, the training of GI professionals remains a top priority to further the application of integrated multidisciplinary care models.

This Special Issue, “Integrated Multidisciplinary Care for Pediatric Inflammatory Bowel Disease: Supporting Disease and Psychosocial Outcomes,” is dedicated to describing the evidence for, and application of, integrated biopsychosocial IBD care. It contains a collection of manuscripts written by expert clinicians and researchers in the field of IBD. We hope that this Special Issue educates others, highlights the importance and value of integrated multidisciplinary care in the field of pediatric IBD, and supports the growth of such programs nationally.

## Data Availability

No new data were created or analyzed in this study. Data sharing is not applicable to this article.
